# Extracellular Vesicles Biogenesis, Cargo Sorting and Implications in Disease Conditions

**DOI:** 10.3390/cells12020280

**Published:** 2023-01-11

**Authors:** Pamali Fonseka, Suresh Mathivanan

**Affiliations:** Department of Biochemistry, La Trobe Institute for Molecular Science, La Trobe University, Bundoora, VIC 3086, Australia

Extracellular vesicles (EVs) are small packages that contain proteins, lipids and nucleic acids and are released by various cell types [1,2]. With the discovery of EVs as mediators of cell-to-cell communication, there is significant research interest in the biogenesis, secretion and functional roles of EVs [3]. EVs are heterogenous by size as well as their performed functions [4,5,6]. EVs have been isolated from a plethora of cells, tissues and biological fluids such as blood, saliva, urine and milk [7,8,9,10]. Despite the growing knowledge of the molecular mechanisms involved in the biogenesis of EVs, cargo sorting and their roles in various diseases, comprehensive biochemical and functional studies are required to unveil the underlying types of machinery in biogenesis in host cells and uptake in recipient cells. This Special Issue focuses on recent findings pertaining to EV biogenesis, cargo sorting in EVs and their role in disease progression.

EVs are known to mirror the host cells by carrying a variety of molecular cargo such as nucleic acids, lipids, proteins and metabolites [11]. Upon the uptake of these molecules by the recipient cells, a cascade of signalling pathways is initiated, which in turn, induces many pathophysiological changes in the recipient cells. The complex mechanisms by which these molecules are sorted into EVs are discussed in this Special Issue. The review by Sherman et al. highlights the ESCRT-dependent and independent mechanisms of protein sorting and the involvement of post-translational modifications (PTMs), RNA-induced silencing complexes (RISCs) and Dicer components in RNA sorting into EVs [12]. Whereas, the Mir and Goettsch group summarises the specific modulator motifs governing the EV cargo packaging and how alterations to these motifs can be used as a potential therapeutic strategy in cardiovascular diseases [13].

Among the many molecules that are incorporated into EVs that are highly implicated in cancer progression, the epidermal growth factor receptor (EGFR) has taken center stage in recent years [14]. This receptor is now known to influence several EV biogenesis pathways, and the reviews by Zanetti-Domingues et al. extensively discuss the mechanisms by which EGFR is incorporated into EVs and the interplay between EGFR signalling and EV biogenesis. Understanding the mechanisms that are involved in the biogenesis, cargo sorting and secretion of EVs can aid in employing EVs in clinical applications [15,16]. In order to keep cellular homeostasis in balance, cells are known to secret more EVs when under lysosomal stress. A pulsed stable isotope labelling of amino acids in cell culture (pSILAC)-based quantitative proteomics study, conducted by Tan et al., has shown the preferential localization of the newly synthesized proteins into the EVs over lysosome hypothalamic cells. This study highlights the importance of cathepsin proteins during EV secretion and in neurological disorders governed by energy homeostasis [17].

The cargo content of EVs often differs with the pathophysiological conditions of the host cells. For instance, the EVs that are isolated from cigarette smoke condensate-treated human small airway epithelial (SAE) cells have a diverse RNA signature compared to the control human airway epithelial cell (AEC)-derived EVs. Corsello et al. were able to identify novel miRNAs that could play a role as potential biomarkers for the diagnosis of cigarette smoke-related diseases [18]. However, when using EVs derived from patient samples in biomarker studies, Newman et al. have shown the importance of subject variability and EV abundance in the collected clinical samples [19].

The tumour microenvironment (TME) consists of a heterogeneous population of cells that secretes rich cargo-containing EVs, which in turn, can mediate phenotypical changes in the cells within TME and distant sites [4,5]. This crosstalk between cancer cells and neighbouring cells often leads to the transfer of drug resistance and activation of immuno-suppressive pathways. Santos et al. have shown the importance of EV miRNA interactions and signalling in the transfer of chemoresistance and survivability [20]. Moreover, EVs have also been shown to promote aggressive phenotype in hepatocellular carcinoma (HCC) by communicating between HCC cells and surrounding adipocytes [21]. During this malignancy, EVs are known to activate a plethora of signalling cascades, including induction of epithelial–mesenchymal transition (EMT), activation of β-catenin signalling, Notch signalling and Smad2/3 signalling pathway [22].

EVs also have recently gained interest as modulators of inflammation and cell death during conditions such as sepsis. The comprehensive review by Sanwlani et al. has summarised types of EVs known to have roles in mediating immune responses leading to cell death and the role of EVs in lung inflammatory disorders [23]. Due to the structure of EVs, they are considered excellent cargo- and drug-carrying vehicles. However, extreme caution must be taken when selecting the source of EVs to be employed as therapeutic vehicles. Gomzikova et al. performed an in-depth analysis of cytochalasin B-induced membrane vesicles (CIMVs) derived from human mesenchymal stem/stromal cells (MSCs) and have shown that their vesicles can be used as cell-free therapy of degenerative diseases [24]. Whereas, a study from the Van Deun group has been shown as a proof concept that functionalising gold nanoparticles by cloaking them with EV membranes can avoid undesirable elimination by immune cells and can improve autologous uptake. Hence, these bioengineered vesicles can be used as drug-delivery systems in the future [25].

EVs also play an important role in cross-species communication. In recent years, there is an exponential increase in studies that have exploited the anti-cancer properties of bovine milk-derived extracellular vesicles (MEVs) [26]. Fonseka et al. have demonstrated that when aggressive neuroblastoma cells are treated with MEVs there is an enrichment of proteins implicated in senescence and apoptosis and a depletion of proteins involved in cell growth. Moreover, these cells were sensitised to doxorubicin treatment in the presence of MEV [27]. EVs derived from various plants also have been shown to play a role in pathophysiological conditions in humans. For instance, Cho et al. have shown that EVs derived from ginseng roots and ginseng cells can improve the replicative senescence of human dermal fibroblasts suggesting the potential use of EVs as a delivery vehicle of bioactive nanomaterials [28]. With the increased interest in fungal EVs that modulate many vital biological functions in fungal cells, more studies are performed to characterise different species of fungi [29]. Karkowska-Kuleta et al. have performed proteomics analysis on EVs produced by the clinically important non-albicans Candida species, which have numerous virulence factors [30].
